# Perspectives on Selected Hemophilia Research Presented at ISTH 2025: Nonfactor Replacement Therapies

**DOI:** 10.1002/ajh.70051

**Published:** 2025-09-15

**Authors:** Annette von Drygalski

**Affiliations:** ^1^ Hematology in San Diego San Diego California USA

1

Please visit https://annenberg.net/courses/61571/PODCAST.html to view the podcast.

This educational activity reviews significant advancements in nonfactor replacement therapies for hemophilia management, addressing limitations of traditional clotting factor concentrates. It highlights key research presented at ISTH 2025, focusing on novel therapeutic approaches. The activity explores the mechanism of action, efficacy, and safety of rebalancing agents and factor VIII mimetics. Discussions include how these agents promote hemostasis through factor‐independent pathways, reduce annualized bleed rates, and offer flexible dosing. Real‐world evidence demonstrates the feasibility of major surgeries for patients on emicizumab with appropriate hemostatic support and the potential for seamless transitions between therapies. The activity addresses the critical aspect of comorbidities, noting the significantly higher prevalence of osteoporosis in patients with severe hemophilia not receiving prophylaxis, underscoring the importance of early intervention.

